# A CT-Based Radiomics Nomogram Integrated With Clinic-Radiological Features for Preoperatively Predicting WHO/ISUP Grade of Clear Cell Renal Cell Carcinoma

**DOI:** 10.3389/fonc.2021.712554

**Published:** 2021-12-03

**Authors:** Yingjie Xv, Fajin Lv, Haoming Guo, Zhaojun Liu, Di Luo, Jing Liu, Xin Gou, Weiyang He, Mingzhao Xiao, Yineng Zheng

**Affiliations:** ^1^ Department of Radiology, The First Affiliated Hospital of Chongqing Medical University, Chongqing, China; ^2^ Department of Urology, The First Affiliated Hospital of Chongqing Medical University, Chongqing, China

**Keywords:** radiomics nomogram, computed tomography, clear cell renal cell carcinoma, WHO/ISUP grading, prediction model

## Abstract

**Objective:**

This study aims to develop and validate a CT-based radiomics nomogram integrated with clinic-radiological factors for preoperatively differentiating high-grade from low-grade clear cell renal cell carcinomas (CCRCCs).

**Methods:**

370 patients with complete clinical, pathological, and CT image data were enrolled in this retrospective study, and were randomly divided into training and testing sets with a 7:3 ratio. Radiomics features were extracted from nephrographic phase (NP) contrast-enhanced images, and then a radiomics model was constructed by the selected radiomics features using a multivariable logistic regression combined with the most suitable feature selection algorithm determined by the comparison among least absolute shrinkage and selection operator (LASSO), recursive feature elimination (RFE) and ReliefF. A clinical model was established using clinical and radiological features. A radiomics nomogram was constructed by integrating the radiomics signature and independent clinic-radiological features. Performance of these three models was assessed using receiver operating characteristic (ROC) curve analysis and decision curve analysis (DCA).

**Results:**

Using multivariate logistic regression analysis, three clinic-radiological features including intratumoral necrosis (OR=3.00, 95% CI=1.30-6.90, p=0.049), intratumoral angiogenesis (OR=3.28, 95% CI=1.22-8.78, p=0.018), and perinephric metastasis (OR=2.90, 95% CI=1.03-8.17, p=0.044) were found to be independent predictors of WHO/ISUP grade in CCRCC. Incorporating the above clinic-radiological predictors and radiomics signature constructed by LASSO, a CT-based radiomics nomogram was developed, and presented better predictive performance than clinic-radiological model and radiomics signature model, with an AUC of 0.891 (95% CI=0.832-0.962) and 0.843 (95% CI=0.718-0.975) in the training and testing sets, respectively. DCA indicated that the nomogram has potential clinical usefulness.

**Conclusion:**

The CT-based radiomics nomogram is a promising tool to predict WHO/ISUP grade of CCRCC preoperatively and noninvasively.

## Introduction

Renal cell carcinoma (RCC) represents the most common malignant neoplasm of the kidney in adults, of which 70–80% are categorized as clear cell renal cell carcinoma (CCRCC) (1, 2). With a continuously increasing incidence for decades, CCRCC is the RCC subtype that accounts for the most metastatic cases and deaths (3–5).

Compared to early-stage CCRCC, advanced CCRCC is considered more aggressive and has a worse prognosis (6). The prognosis of patients with CCRCC is closely related to the tumor nuclear grade (7). As the most generally adopted grading system, the World Health Organization/International Society of Urological Pathology (WHO/ISUP) classification system categorizes tumors of nuclear grade I and II as low-grade and of grade III and IV as high- grade. High-grade CCRCC differs from low-grade CCRCC in malignant biological behaviors that generate mortal clinical outcomes (8). Therefore, it is crucial for clinicians to identify the nuclear differentiation degree of CCRCC because of the important role it plays in formulating a clinical treatment strategy. However, although percutaneous biopsy has been criticized for the risks of procedural complications, potential sampling errors, and mismatch with pathology outcomes, it is the only preoperative method for identifying the confirmed grade of CCRCC (9). A noninvasive, efficient method for identifying the pathological grade of CCRCC is urgently needed.

Computed tomography (CT) is a common noninvasive imaging modality for diagnosing tumor staging and assessing tumor aggressiveness in patients with CCRCC (4). Nonetheless, CT has limited predictive performance in differentiating high-grade from low-grade CCRCCs, and the accuracy of diagnosis based on CT images depends on the experience of radiologists to a great extent, which is extremely subjective (10). Radiomics, a promising and emerging technique, enables the conversion of medical images into enormous quantities of image-related features which can be analyzed in model-building algorithms (11–13). To date, radiomics has successfully been applied in several fields of RCC, including prediction of the Fuhrman stages and therapy response of CCRCC and discrimination of RCC’s subtypes (14–16). However, most studies developed models based on texture analysis only, which neglected the importance of clinical risk factors and radiological features that could improve predictive performance.

The purpose of this study was to develop and validate a radiomics nomogram that incorporates NP CT radiomics signature, clinical factors, and radiological features to preoperatively differentiate high-grade CCRCC from low-grade CCRCC.

## Materials and Methods

### Patients

**Figure 1:**
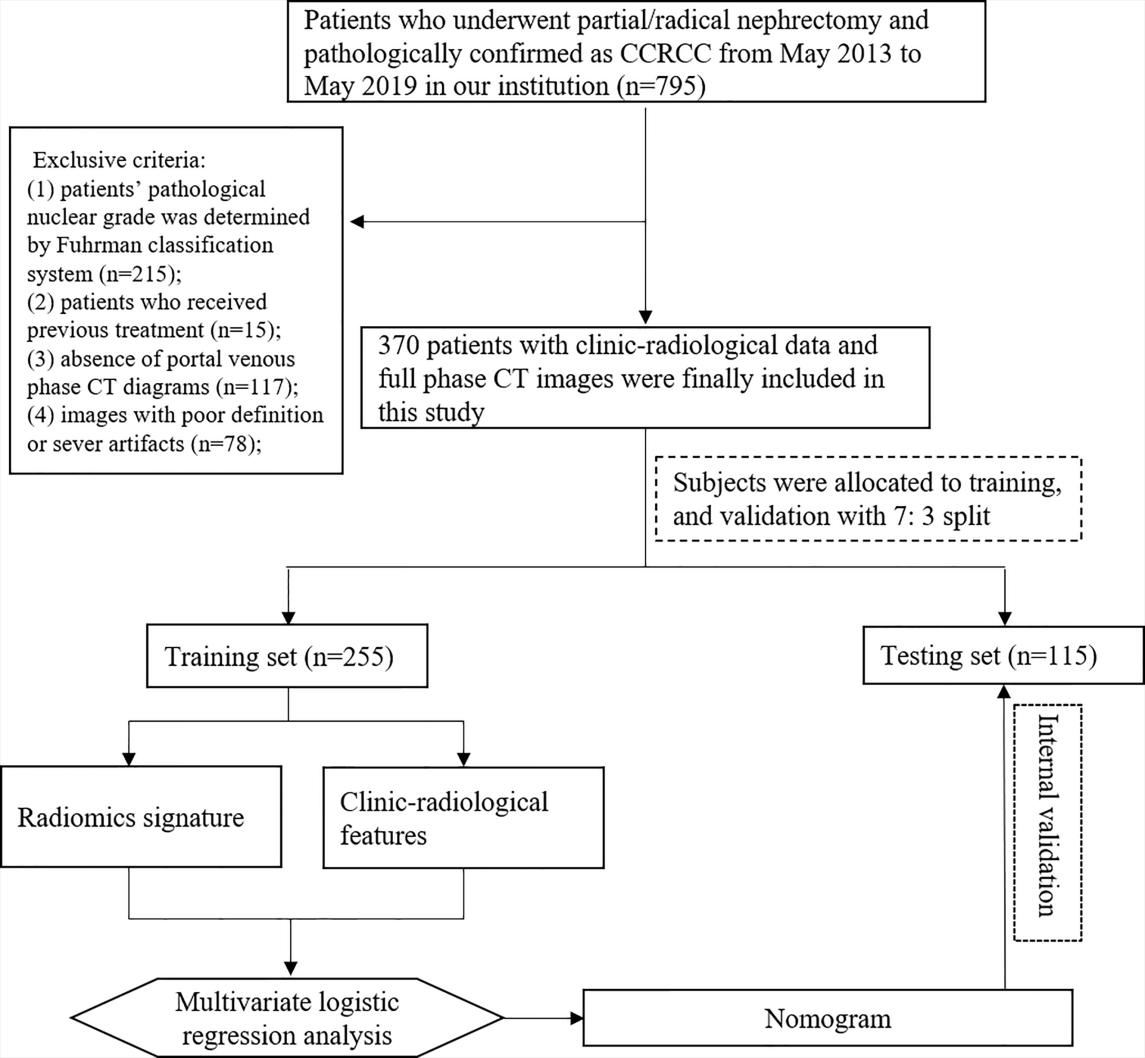
Flowchart of recruiting study population and model construction.

All patients were recruited between May 2013 to May 2019 at the First Affiliated Hospital of Chongqing Medical University. The inclusion criteria were as follows: 1) patients underwent partial/radical nephrectomy and pathologically diagnosed as CCRCC; 2) availability of complete clinical, pathological, and CT image data. A total of 795 patients met the inclusion criteria were initially enrolled. We excluded patients whose CCRCC nuclear grades were determined by the Fuhrman classification system (n=215), and those received preoperative treatment (n=15). Patients without portal venous phase CT images or with the images in poor definition were also excluded (n=195). Finally, 370 patients were retained and allocated to the training (n=255) and testing (n=115) sets. The flowchart of recruiting the patient cohort is shown in **Figure 1**. Two independent pathologists rechecked the CCRCC samples of our study population and reported the histopathological nuclear grade based on the 2016 WHO/ISUP nuclear grading system. Discordant reports were resolved by a third senior pathologist. These tumors were divided into low-grade (grade I and II) and high-grade (grade III and IV). Ethics approval of the institutional review board of our hospital was achieved before the conduction of all protocols and the requirement for obtaining informed consent was waived in this retrospective study.

### CT Imaging Parameters

The routine abdominal CT scanning were acquired using a 64-slice multidetector CT equipment (Discovery 750 HD, GE Healthcare, Milwaukee, WI). The scanning parameters were 120-140 kVp tube voltage, 220–300 mAs tube current, 64 × 0.625 mm detector collimation, matrix of 512 × 512, gantry rotation time of 0.5 s and slice thickness of 5 mm. An iodinated nonionic contrast agent dosed at 1 mL/kg body weight was injected into the antecubital vein at 2.5-3.0 mL/s using an electric power injector. Pre-contrast CT of the abdomen was first acquired, followed by two post-contrast CT scans obtained in corticomedullary phase (CMP, 25-28 s after contrast agent was administrated) and nephrographic phase (NP, 65-70 s after contrast agent was administrated). Finally, excretory phase was acquired (EP, 6-8 min after contrast agent was administrated) (17–19).

### Tumor Segmentation

All NP phase CT images in DICOM format with original dimensions and resolution were transferred into the ITK-SNAP software (version 3.8, www.itksnap.org) for three-dimensional (3-D) segmentation of the region of interests (ROIs). To ensure the accuracy of the tumor boundaries, the ROIs were meticulously manually delineated on all slices, by a radiologist with 10 years of experience in abdominal imaging, who was blinded to the pathological results (reader 1). To test feature stability, radiomics features of 30 randomly chosen patients (from the whole study set) were re-extracted by reader 1 and another radiologist (with 15 years of experience; reader 2). The intraclass correlation coefficient (ICC) was calculated to evaluate the consistency and reproducibility of the features. Features with ICC>0.80 in both intra- and inter-observer agreement analyses were included in subsequent analysis. To avoid partial volume effect, the top and bottom layers were excluded.

### Radiomics Feature Extraction

The following image preprocessing steps was performed to decrease the feature variability prior to radiomics feature extraction, including gray-level discretization, intensity normalization and voxel resampling. Then, radiomics features were extracted from NP CT images *via* an open-source PyRadiomics library, and classified into four categories: size and morphological features, descriptors of the image intensity histogram, descriptors of the relationships between image voxels (e.g. gray-level co-occurrence matrix (GLCM), run length matrix (RLM), size zone matrix (SZM), and neighborhood gray tone difference matrix (NGTDM) derived textures), and higher-order texture features extracted from filtered images.

### Radiomics Signature Construction

Radiomics features with ICCs greater than 0.80 in the agreement analysis were reserved. Next, three feature selection algorithms including least absolute shrinkage and selection operator (LASSO), recursive feature elimination (RFE) and ReliefF were applied to select the optimized subset of features for radiomics model construction, respectively, which was used as the input fed into multivariable logistic regression. Third, the predictive performance of the three prediction models were compared and the one with top performance was retained. The selected radiomics features were used to construct radiomics signature.

### Clinic-Radiological Model Building

**Figure 2:**
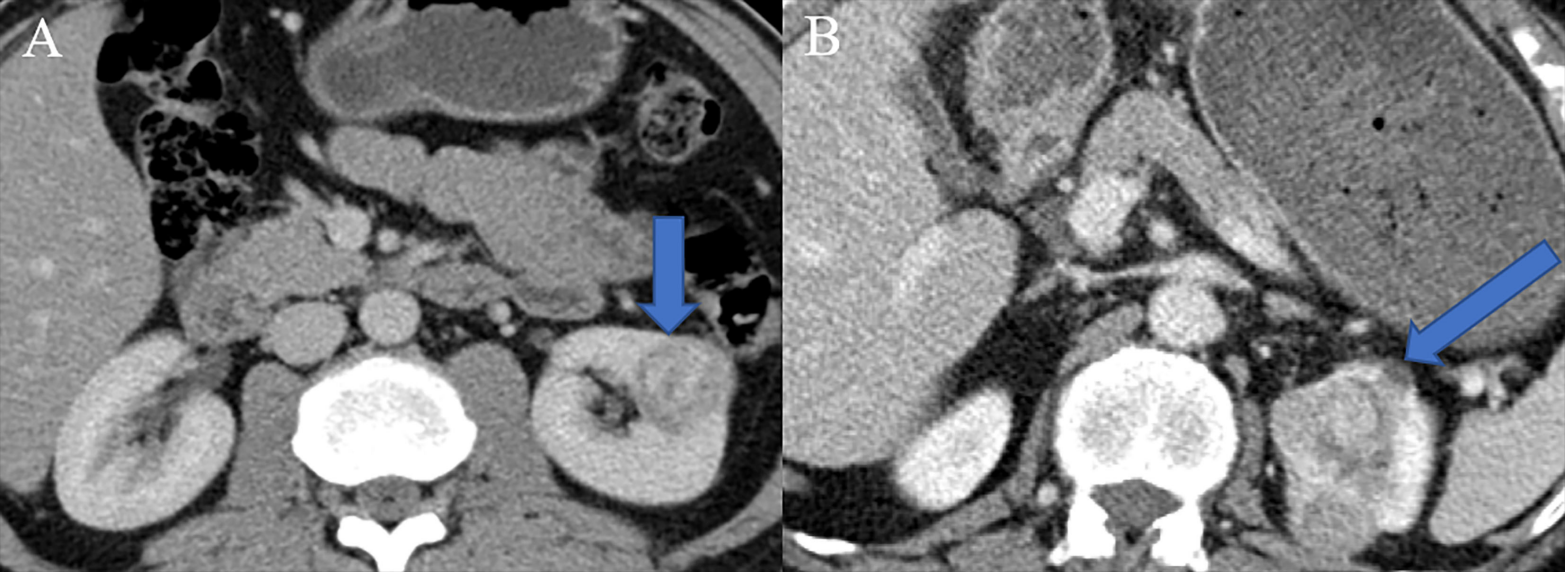
Two representative nephrographic phase CT images. **(A)** A patient with low-grade CCRCC, there was no specific radiological features in the presented CT image. **(B)** A patient with high-grade CCRCC, tumor necrosis, angiogenesis, and perinephric invasion phenomena were observed in the presented CT image.

Clinical parameters such as age, sex, body mass index (BMI), smoking history, hypertension history, diabetes history, tumor location, specific clinical symptoms (hematuria and flank pain), and distant metastasis were retrieved from the electronic medical record system of our institution. The radiological features including tumor size, intratumoral necrosis, cystic degeneration, intratumoral calcification, invasion of the renal capsule, intratumoral angiogenesis, venous invasion, and perinephric metastasis were reviewed and reported by two radiologists with 10 and 15 years of experience in abdominal imaging who were blinded to the radiological reports and pathologic details. The diagnostic criteria were summarized in **Table S1**. For the construction of the clinic–radiological model, the univariate regression was firstly applied to analyze the above clinic-radiological features, and the statistically significant features in the univariate regression analysis were then processed in the multivariate regression model. Finally, features with a P-value lower than 0.05 were adopted to establish clinic–radiological model. Two representative nephrographic phase CT images in which one was low-grade CCRCC (**Figure 2A**) and another was high-grade (**Figure 2B**) were exhibited in **Figure 2**.

### Development of Radiomics Nomogram

To provide the clinician with a quantitative tool to discriminate high-grade from low-grade CCRCCs, radiomics signature and clinic-radiological characteristics were combined by multivariable logistic regression analysis to construct a radiomics nomogram model as the combined model.

### Model Evaluation

5-fold cross validation was used in model training, and the diagnostic performance of clinic-radiological, radiomics, and combined models were validated in terms of the receiver operating characteristic (ROC) curve and area under the curve (AUC) in a testing set. Sensitivity, specificity, positive predictive value (PPV), negative predictive value (NPV), and accuracy were also calculated. The Delong test was used to compare the AUC values in different models in both training and testing sets. The calibration curve analysis was performed to determine the predictive performance of the nomogram in the testing set, accompanied with the Hosmer-Lemeshow test. Decision curve analysis (DCA) was also performed to assess the clinical significance of the radiomics nomogram by calculating the net benefits at different threshold probabilities.

### Statistical Analysis

Continuous variables are expressed as mean values ± standard deviations and categorical variables are expressed as counts (n) and percentages (%). Normally distributed continuous data were compared using the student’s t-test. The Chi-square test was used to compare the distribution of categorical data between groups. The multivariate logistic regression analysis was applied to determine the independent predictors among all the clinical variables. All statistical analyses were performed using R software (version 3.5.2). A two-tailed P-value lower than 0.05 was considered statistically significant.

## Results

### Clinic-Radiological Characteristics

A total of 370 patients were enrolled in our study, with collected clinical and radiological data. The differences in clinic-radiological variables between patients with low- and high-grade CCRCCs in the training and testing sets are summarized in **Table 1**. The training set included 255 patients (152 males and 103 females), in which 202 patients were diagnosed with low-grade CCRCC while 53 were diagnosed with high-grade one. Patients with high-grade CCRCC were significantly different from those with low-grade CCRCC in terms of tumor size (p<0.001), surgical method (p<0.001), intratumoral necrosis (p<0.001), intratumoral calcification (p=0.027), invasion of the renal capsule (p<0.001), intratumoral angiogenesis (p<0.001), venous invasion (p<0.001), and perinephric metastasis (p<0.001).

**Table 1:** Clinic-radiological characteristics of CCRCC patients in the training and testing sets.

Characteristics		Training set (n = 255)			Testing set (n = 115)		
		Low-grade	High-grade	*P* value	Low-grade	High-grade	*P* value
Full cohort, n (%)	370	202 (79.22%)	53 (20.78%)	–	94 (81.74%)	21 (18.26%)	–
Age (Y)		56.93 ± 11.64	60.19 ± 11.14	0.068	58.18 ± 12.46	56.24 ± 16.53	0.55
Sex, n (%)	male	118 (58.4%)	34 (64.2%)	0.45	50 (53.2%)	17 (81.0%)	0.02
	female	84 (41.6%)	19 (35.8%)		44 (46.8%)	4 (19.0%)	
BMI (kg/m²)		24.34 ± 3.50	23.98 ± 4.58	0.54	24.96 ± 5.13	22.80 ± 3.02	0.07
Smoking history, n (%)		61 (30.2%)	22 (41.5%)	0.12	35 (37.2%)	7 (33.3%)	0.74
Hypertension, n (%)		76 (37.6%)	15 (28.3%)	0.21	41 (43.6%)	6 (28.6%)	0.21
Diabetes, n (%)		28 (13.9%)	8 (15.1%)	0.82	19 (20.2%)	4 (19.0%)	0.90
Tumor size (cm)		4.1 ± 1.92	5.89 ± 2.89	<0.001	4.31 ± 2.20	5.87 ± 2.88	0.03
Tumor location, n (%)	left	108 (53.5%)	22 (41.5%)	0.12	52 (55.3%)	13 (61.9%)	0.58
	right	94 (46.5%)	31 (58.5%)		42 (44.7%)	8 (38.1%)	
Surgical Method, n (%)	partial	119 (58.9%)	13 (24.5%)	<0.001	47 (50.0%)	6 (28.6%)	0.08
	radical	83 (41.1%)	40 (75.5%)		47 (50.0%)	15 (71.4%)	
Hematuria, n (%)		22 (10.9%)	9 (17.0%)	0.23	11 (11.7%)	6 (28.6%)	0.10
Flank pain, n (%)		26 (12.9%)	9 (17.0%)	0.44	16 (17.0%)	5 (23.8%)	0.47
Distant Metastasis, n (%)		0 (0.0%)	1 (1.9%)	0.21	0 (0.0%)	1 (4.8%)	0.18
Intratumoral Necrosis, n (%)		82 (40.6%)	41 (77.4%)	<0.001	36 (38.3%)	15 (71.4%)	0.006
Cystic Degeneration, n (%)		23 (11.4%)	5 (9.4%)	0.69	9 (9.6%)	0 (0.0%)	0.30
Intratumoral Calcification, n (%)		8 (4.0%)	7 (13.2%)	0.027	2 (2.1%)	4 (19.0%)	0.009
Invasion of the Renal Capsule, n (%)		22 (10.9%)	19 (35.8%)	<0.001	7 (7.4%)	10 (47.6%)	<0.001
Intratumoral Angiogenesis, n (%)		116 (57.4%)	46 (86.8%)	<0.001	56 (59.6%)	19 (90.5%)	0.007
Renal vein invasion, n (%)		1 (0.5%)	6 (11.3%)	<0.001	0 (0.0%)	1 (4.8%)	0.18
Perinephric Metastasis, n (%)		13 (6.4%)	17 (32.1%)	<0.001	4 (4.3%)	7 (33.3%)	<0.001

### Clinic-Radiological Model Building

Univariate analysis showed that tumor size, intratumoral necrosis, intratumoral calcification, invasion of the renal capsule, intratumoral angiogenesis, venous invasion, and perinephric metastasis served as the risk factors of WHO/ISUP grade in CCRCC. After multivariate logistic regression analysis, intratumoral necrosis (OR=3.00, 95% CI=1.30-6.90, p=0.049), intratumoral angiogenesis (OR=3.28, 95% CI=1.22-8.78, p=0.018), and perinephric metastasis (OR=2.90, 95% CI=1.03-8.17, p=0.044) remained to be independent clinic-radiological predictors (**Table 2**).

**Table 2:** Univariate and multivariate logistic regression analysis of the clinic-radiological features in predicting the WHO/ISUP grade of CCRCC.

Characteristics	Univariate analysis			Multivariate analysis		
	OR	95% CI	*P* value	OR	95% CI	*P* value
Tumor size	1.37	1.20-1.56	<0.001	0.99	0.82-1.19	0.88
Operative Method	4.41	2.22-8.76	<0.001	1.71	0.73-3.99	0.21
Intratumoral Necrosis	5.00	2.48-10.09	<0.001	3.00	1.30-6.90	0.049
Intratumoral Calcification	3.69	1.27-10.70	0.016	2.78	0.78-9.92	0.12
Violation of the Renal Capsule	4.57	2.24-9.35	<0.001	1.22	0.47-3.17	0.68
Intratumoral Angiogenesis	4.87	2.10-11.32	<0.001	3.28	1.22-8.78	0.018
Renal vein invasion	25.66	3.02-218.24	0.003	6.38	0.56-72.68	0.14
Perinephric Metastasis	6.87	3.07-15.36	<0.001	2.90	1.03-8.17	0.044

### Radiomics Feature Selection and Radiomics Model Construction

**Figure 3:**
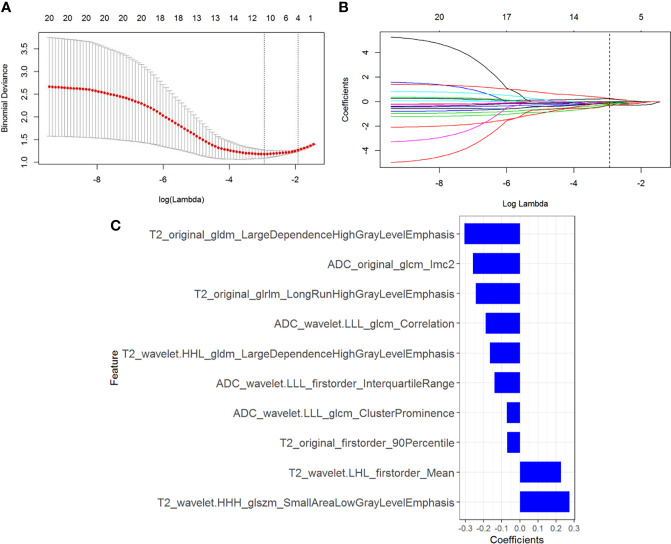
Radiomics feature selection using the least absolute shrinkage and selection operator (LASSO) regression. **(A)** Tuning parameter (λ) selection in the LASSO model. The optimal value of λ= 0.053, with log(λ) =-2.937 was selected. **(B)** LASSO coefficient profiles of the N radiomics features. A coefficient profile plot was generated *versus* the selected log (λ) value with 5-fold cross validation. **(C)** The selected radiomics features (with nonzero coefficients) and their coefficients.

In total, 1320 radiomics features were extracted from NP CT images of each CCRCC patients, among which 480 features with good reproducibility were selected for radiomics model establishment. Three feature selection algorithms such as LASSO, RFE, and ReliefF were applied and compared for dimensionality reduction, and the different feature subsets were fed into logistic regression model for differentiating high-grade from low-grade CCRCCs, respectively. The classification performance for different feature selection algorithms was presented in **Table 3**. It was observed that 10 optimized features were selected by the LASSO algorithm to construct the radiomics model (**Figure 3**), which outperformed than others constructed by RFE and ReliefF and yielded an AUC value of 0.833 (95% CI=0.751-0.925) and 0.804 (95% CI=0.667-0.958) in the training and testing sets, respectively. A radiomics signature was calculated, based on the weighting coefficients of the selected features, using the formula as follows:

**Table 3:** Predictive performance of three feature selection algorithms.

Model	LASSO		RFE		ReliefF	
	Training	Testing	Training	Testing	Training	Testing
AUC (95% CI)	0.833 (0.751-0.925)	0.804 (0.667-0.958)	0.784 (0.609-0.925)	0.742 (0.560-0.897)	0.814 (0.703-0.919)	0.771 (0.623-0.940)
Accuracy	0.778 (0.678-0.859)	0.783 (0.618-0.902)	0.717 (0.598-0.887)	0.662 (0.513-0.851)	0.742 (0.624-0.920)	0.698 (0.543-0.874)
Sensitivity	0.854	0.850	0.801	0.762	0.838	0.823
Specificity	0.691	0.706	0.634	0.598	0.678	0.639
PPV	0.759	0.773	0.646	0.602	0.687	0.632
NPV	0.806	0.803	0.739	0.896	0.774	0.715

Rad-score = -0.244*T2_original_glrlm_LongRunHighGrayLevelEmphasis+-0.188*ADC_wavelet.LLL_glcm_Correlation+-0.166*T2_wavelet.HHL_gldm_LargeDependenceHighGrayLevelEmphasis+-0.072*ADC_wavelet.LLL_glcm_ClusterProminence+-0.069*T2_original_firstorder_90Percentile+-0.26*ADC_original_glcm_Imc2+0.227*T2_wavelet.LHL_firstorder_Mean+0.275*T2_wavelet.HHH_glszm_SmallAreaLowGrayLevelEmphasis+-0.14*ADC_wavelet.LLL_firstorder_InterquartileRange+-0.305*T2_original_gldm_LargeDependenceHighGrayLevelEmphasis + 0.101.

### Radiomics Nomogram Construction

**Figure 4:**
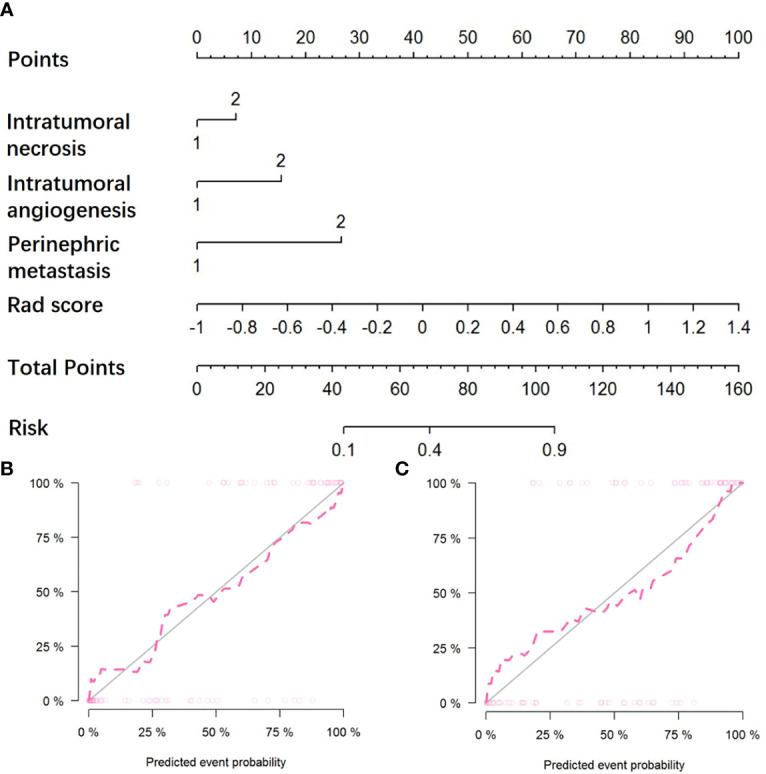
The CT-based radiomics nomogram and calibration curves of the nomogram. **(A)** Integrating radiomics signature, intratumoral necrosis, intratumoral angiogenesis, and perinephric metastasis, the CT-based nomogram was established. Calibration curves of the nomogram in the training **(B)** and testing **(C)** sets.

By incorporating three independent clinic-radiological factors such as intratumoral necrosis, intratumoral angiogenesis, and perinephric metastasis, a combined model was constructed and presented as a CT-based radiomics nomogram (**Figure 4A**). Using the calibration curve analysis, a good agreement between the predicted and actual probabilities for predicting the WHO/ISUP grade of CCRCC in the training and testing sets was illustrated (**Figures 4B, C**). The Hosmer-Lemeshow test yielded a nonsignificant statistical difference (P =0.397 and 0.302).

### Comparison Among Different Models

**Figure 5:**
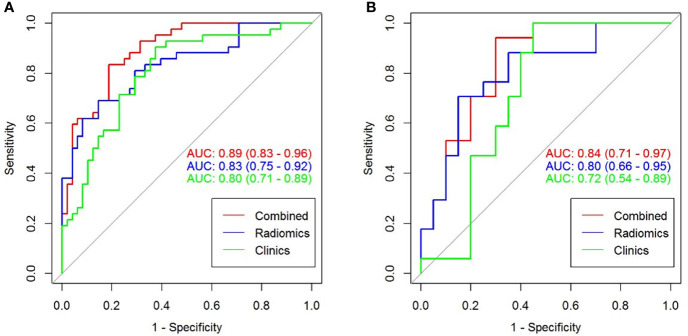
The ROC curves (AUC) of the three models in the training **(A)** and testing sets **(B)**.

**Figure 6:**
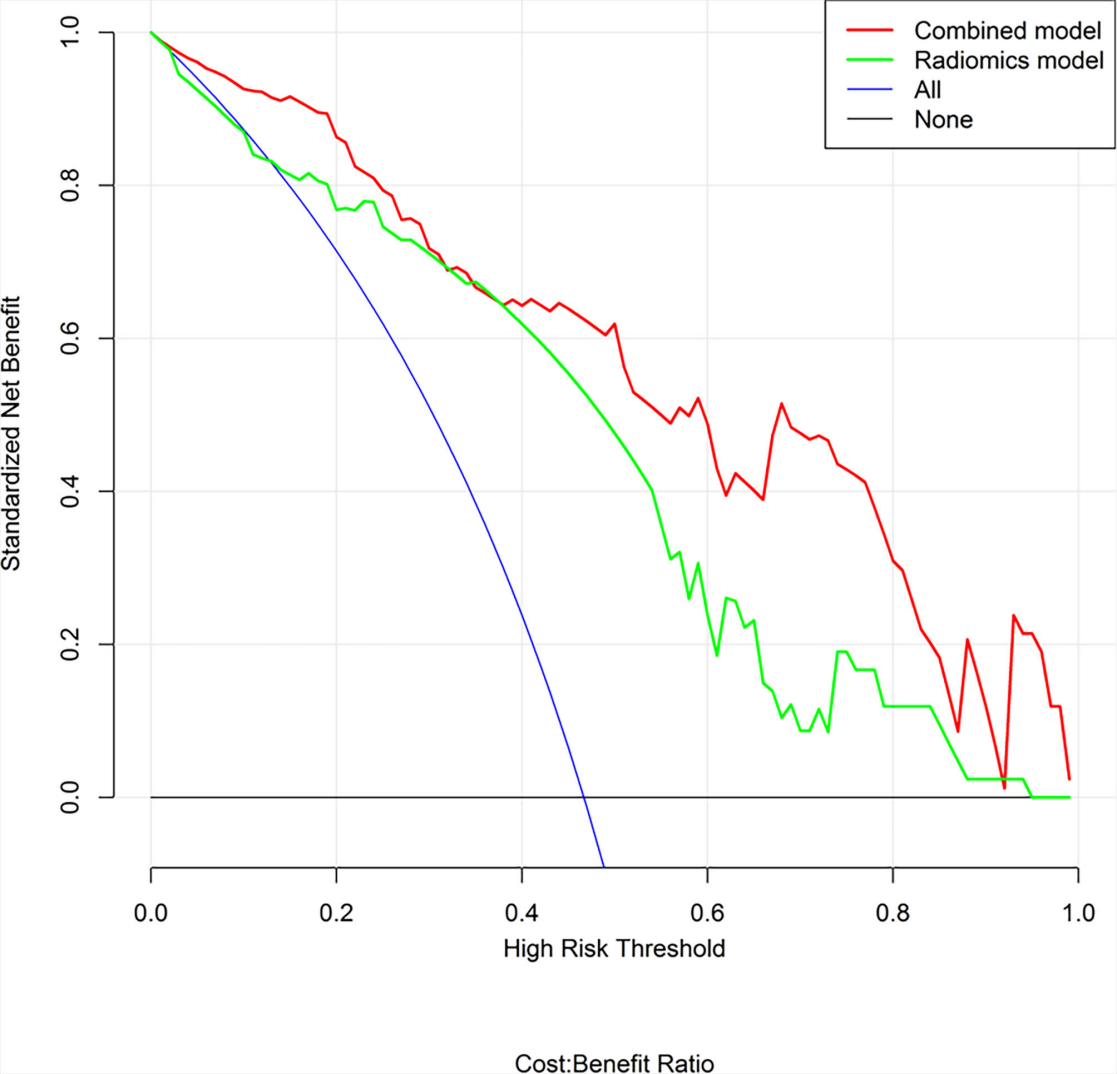
Decision curve analysis (DCA) for the radiomics nomogram and radiomics model. The DCA indicated that more net benefits within the most of thresholds probabilities were achieved using the radiomics nomogram.

The predictive performance and the ROC curves of the clinic-radiological, radiomics signature, and nomogram in the training and testing sets are presented in **Table 4** and **Figure 5**. The clinic-radiological model yielded an AUC value of 0.809 (95% CI=0.715-0.897) in the training set and 0.722 (95% CI=0.546-0.894) in the testing set, while the radiomics signature model obtained an AUC value of 0.833 (95% CI=0.751-0.925) and 0.804 (95% CI=0.667-0.958) in both sets. The nomogram achieved the best discrimination in the training (AUC, 0.891; 95% CI, 0.832-0.962) and testing (AUC, 0.843; 95% CI, 0.718-0.975) sets, with accuracy of 0.822 and 0.811, sensitivity of 0.796 and 0.727, and specificity of 0.848 and 0.933, respectively. Using the Delong test, significant differences between the clinic-radiological model and the CT-based radiomics nomogram with respect to AUC were demonstrated for the training (p =0.003) and testing (p < 0.001) sets. The DCA was presented in **Figure 6**. The radiomics nomogram demonstrated the higher overall net benefit than radiomics model, indicating the radiomics nomogram had an excellent clinical utility in distinguishing high-grade from low-grade CCRCCs.

**Table 4:** Predictive performance of clinic-radiological model, radiomics signature, and radiomics nomogram.

Model	Radiomics nomogram		Radiomics signature		Clinic-radiological model	
	Training	Testing	Training	Testing	Training	Testing
AUC (95% CI)	0.891 (0.832-0.962)	0.843 (0.718-0.975)	0.833 (0.751-0.925)	0.804 (0.667-0.958)	0.809 (0.715-0.897)	0.722 (0.546-0.894)
Accuracy	0.822 (0.727-0.895)	0.811 (0.649-0.920)	0.778 (0.678-0.859)	0.783 (0.618-0.902)	0.756 (0.654-0.840)	0.703 (0.530-0.841)
Sensitivity	0.796	0.727	0.854	0.850	0.679	0.636
Specificity	0.848	0.933	0.691	0.706	0.882	0.801
PPV	0.833	0.941	0.759	0.773	0.905	0.824
NPV	0.813	0.709	0.806	0.804	0.625	0.621

## Discussion

In this retrospective study, we developed and validated a radiomics nomogram for noninvasive, and individualized prediction of WHO/ISUP nuclear grade of CCRCC. The nomogram incorporates clinic-radiological characteristics and NP CT radiomics signature, which demonstrated impressive predictive efficiency (AUC=0.84, 95% CI=0.71-0.97) in stratifying the WHO/ISUP grading levels of CCRCC patients with satisfactory reproducibility and reliability.

The nuclear grade of CCRCC is correlated with metastatic potential and affects patient prognosis (4, 7). Therefore, the preoperative prediction of the nuclear grade is of great significance for clinical decisions. CT diagnosis is superior to percutaneous biopsy because of its noninvasiveness; however, it is inferior in terms of diagnostic accuracy (20, 21). Novel imaging techniques, such as magnetic resonance imaging (MRI) and dual-energy spectral CT, are capable to assess the grading level of CCRCC, however, their predictive performance do not match that of percutaneous biopsy, and the diagnostic results depend on the experience of radiologists (22–24). By quantifying tumor heterogeneity through the spatial arrangement of image voxels with signal-intensity variations and detecting the imperceptible differences of the intensity distribution in medical images, CT radiomics can noninvasively predict the pathological grade of tumors with satisfactory performance (25–27). To our knowledge, no previous study has predicted the WHO/ISUP grade of CCRCC using the combination of NP CT-based radiomics features and clinic-radiological characteristics.

Several studies have demonstrated that machine learning (ML)-based CT radiomics models can distinguish Fuhrman grade or WHO/ISUP grade of CCRCC (17, 19, 28–30). However, most of these studies built ML models based on radiomics features only, neglecting the importance of clinical and radiological characteristics (17, 19). The radiomics-derived data are not a panacea for computerized clinical decision-support systems. Our study summarized the imperceptible distinctions of CCRCC patients’ clinic-radiological characteristics and CT radiomics features and analyzed them using a nomogram. In terms of clinical information, we chose to investigate several indicators that can be independent predictors for WHO/ISUP grade. Regarding radiological data, we concentrated on features such as necrosis, tumor microvessels, early metastasis, and tiny calcification, which may indicate a poor prognosis, and display different density and texture on CT images. Previous reports have shown that CCRCC is a highly angiogenic and vascularized tumor (31). In addition, the differences in enhancement patterns between low- and high-grade CCRCCs have been proved to correlate to the hemodynamics and microvessel density (MVD) of individual RCC lesions (32, 33). Ficarra et al. (34) validated that intratumoral necrosis as a prognostic factor is useful in the clinical management of CCRCC patients, which is recommended by the Mayo Clinic Stage, Size, Grade, and Necrosis (SSIGN) scoring system (35). Our results were consistent with former researches, as intratumoral necrosis (OR=3.00, 95% CI=1.30-6.90, p=0.049), intratumoral angiogenesis (OR=3.28, 95% CI=1.22-8.78, p=0.018), and perinephric metastasis (OR=2.90, 95% CI=1.03-8.17, p=0.044) were proved to be independent factors of high-grade CCRCC and were used to establish a clinic-radiological model.

Contrast-enhanced CT examination revealed different information as time progressed. To the best of our knowledge, features extracted from full-phase or CMP combined with NP images are the most common objects of the ML-based radiomics model to predict the Fuhrman or WHO/ISUP grade of CCRCC (19, 28, 36, 37). Huhdanpaa et al. (38) found that absolute enhancement and residual enhancement in the NP phase are both more heterogeneous for low-grade tumors. As a result, even though the predictive performance of based NP CT-based model may decline compared with that using the full-phase CT images, the effectiveness of nomogram based on NP CT images alone has been explored first for predicting the WHO/ISUP grade of CCRCC. Surprisingly, the prediction model we built reached a relatively superior performance compared to the relevant studies (AUC=0.843, 95% CI, 0.718-0.975; accuracy=0.811, sensitivity=0.727, and specificity=0.933), and this could be attributed to the comparatively large sample size and aggregation of a variety of features in our study.

To explore clinical use, further incorporating the radiomics signature, intratumoral necrosis, intratumoral angiogenesis, and perinephric metastasis, an easy-to-use CT-based radiomics nomogram was constructed, which could preoperatively distinguish high-grade from low-grade CCRCCs and facilitate personalized treatment decisions. With AUCs of 0.891 (95% CI, 0.832-0.962) and 0.843 (95% CI, 0.718-0.975) in the training and testing set, respectively, the nomogram achieved an improved predictive performance compared with the radiomics model or clinic-radiological model alone. Moreover, DCA was performed to assess the overall net benefit of the nomogram. As a result, more net benefits within the most of thresholds probabilities were achieved using the radiomics nomogram, indicating that using the nomogram may obtain a better clinical outcome in formulating therapy strategies. Therefore, the CT-based radiomics nomogram can be regarded as a promising assistive tool to preoperatively differentiate high-grade from low-grade CCRCCs.

This study is limited to several conditions. First, as a single-center retrospective study, it might be subject to inherent biases and unknown confounders that make it less generalizable to other institutions. Second, the exact tumor region deviates from manual tumor segmentation in some tiny areas, which may result in absence of necessary features in the tumor edge; as such, an automated segmentation tool with accurate tumor identification ability is highly anticipated. Third, manual tumor segmentation was time-consuming, and automatic segmentation methods should be developed in the future.

In conclusion, this study proposed and validated a CT-based radiomics nomogram that integrating with clinic-radiological predictors and radiomics signature, which demonstrated an excellent predictive ability in differentiating high-grade from low-grade CCRCCs. As a non-invasive, preoperative method, the radiomics nomogram may facilitate patient stratification and clinical decision making for the patients with CCRCC.

## Data Availability Statement

The raw data supporting the conclusions of this article will be made available by the authors, without undue reservation.

## Ethics Statement

The studies involving human participants were reviewed and approved by the institutional review board of the First Affiliated Hospital of Chongqing Medical University. The patient informed consent was waived for the retrospective usage of patients’ medical images.

## Author Contributions

MX, YZ, and FL conceived the project. YX and YZ analysed the data and wrote the paper. YX, ZL, DL and HG collected the data. JL, XG and WH provided clinical expertise. All authors contributed to the article and approved the submitted version.

## Funding

The project was funded by the Chongqing municipal health and Health Committee (CQYC2020020318).

## Conflict of Interest

The authors declare that the research was conducted in the absence of any commercial or financial relationships that could be construed as a potential conflict of interest.

## Publisher’s Note

All claims expressed in this article are solely those of the authors and do not necessarily represent those of their affiliated organizations, or those of the publisher, the editors and the reviewers. Any product that may be evaluated in this article, or claim that may be made by its manufacturer, is not guaranteed or endorsed by the publisher.
