# Supplementary material for: A CT-Based Radiomics Nomogram Integrated With Clinic-Radiological Features for Preoperatively Predicting WHO/ISUP Grade of Clear Cell Renal Cell Carcinoma
Source: Front Oncol. 2021 Dec 3;11:712554. doi: 10.3389/fonc.2021.712554 (PMC8677659; doi:10.3389/fonc.2021.712554)
Supplement: Supplementary file 1 [file Table_1.docx]

Table S1 Diagnostic criteria of CT imaging features

| Features | Diagnostic criteria |
| --- | --- |
| Tumor size | the maximum diameter on transverse images |
| Intratumoral necrosis | the non-enhanced liquid area of the tumor is more than 50% of the tumor^1^ |
| Cystic degeneration | target lesion showing uniform water density and signal-intensity, and no enhancement on enhancement examination^2^ |
| Intratumoral calcification | obvious dense shadows in the parenchyma, which were speckled, lined, or shell-shaped |
| Violation of the renal capsule | abnormal lesion violating the margin of renal capsule |
| intratumoral angiogenesis | vascular enhancement seen in the parenchyma of the cortical stage tumor^1,3^ |
| Renal vein invasion | imaging features of tumor thrombosis in the renal vein and inferior vena cava^1^ |
| Perinephric metastasis | perinephric invasion that breaks through the renal capsule on the CT imging |

References:

1 Jiang, Y. *et al.* A Computed Tomography-Based Radiomics Nomogram to Preoperatively Predict Tumor Necrosis in Patients With Clear Cell Renal Cell Carcinoma. *Frontiers in oncology* **10**, 592, doi:10.3389/fonc.2020.00592 (2020).

2 Tse, J., Shen, J., Shen, L., Yoon, L. & Kamaya, A. Bosniak Classification of Cystic Renal Masses Version 2019: Comparison of Categorization Using CT and MRI. *AJR. American journal of roentgenology* **216**, 412-420, doi:10.2214/ajr.20.23656 (2021).

3 Meng, X., Shu, J., Xia, Y. & Yang, R. A CT-Based Radiomics Approach for the Differential Diagnosis of Sarcomatoid and Clear Cell Renal Cell Carcinoma. *BioMed research international* **2020**, 7103647, doi:10.1155/2020/7103647 (2020).
